# Fertility-Associated Soil Chemistry Predominantly Influence Gut Microbiota Diversity in Goitered Gazelles of the Qaidam Basin, China

**DOI:** 10.3390/microorganisms14020391

**Published:** 2026-02-06

**Authors:** Qing Zhao, Bin Li, Chengbo Liang, Jiaxin Wei, Juan Ma, Wen Qin

**Affiliations:** 1 State Key Laboratory of Plateau Ecology and Agriculture, Qinghai University, Xining 810016, China; zhaoqing1063@163.com (Q.Z.); weijiaxinyouxiang@163.com (J.W.); 15682920216@163.com (J.M.); 2 School of Ecological and Environmental Engineering, Qinghai University, Xining 810016, China; 3 Qinghai Provincial Key Laboratory of Animal Ecological Genomics, Xining 810001, China; libin@nwipb.cas.cn (B.L.); lcb2690375470@outlook.com (C.L.); 4 Key Laboratory of Adaptation and Evolution of Plateau Biota, Northwest Institute of Plateau Biology, Chinese Academy of Sciences, Xining 810001, China

**Keywords:** *Gazella subgutturosa*, 16S rRNA, alpha and beta diversity, soil properties, source tracking analysis

## Abstract

This study focused on the links between soil physicochemical properties and the gut microbiota of goitered gazelles (*Gazella subgutturosa*) in the hyper-arid Qaidam Basin. By integrating 16S rRNA gene sequencing, soil physicochemical analysis (11 soil indicators), and microbial source tracking (FEAST) on samples of feces (*n* = 58), soil (*n* = 35), and water (*n* = 35) collected from six typical regions. We systematically revealed the mechanisms by which soil properties influence the gut microbiome of wildlife in an arid desert ecosystem based on source tracking and Multiple Regression on distance Matrices (MRM) analysis. The results showed that soil total phosphorus (TP) was significantly positively correlated with the α-diversity of gut microbiota (coefficient = 0.4/0.23/0.332; *p* < 0.05), while soil organic carbon (SOC) was significantly negatively correlated (coefficient = −0.44/−0.436; *p* < 0.05), indicating that soil nutrients indirectly predict host microbial diversity by regulating vegetation productivity and forage quality. β-diversity analysis further demonstrated that spatial heterogeneity in soil pH (coefficient = 0.3083; *p* < 0.05) and TP (coefficient = 0.227; *p* < 0.05) significantly drove the structural differentiation of gut microbial communities. Source-tracking results based on FEAST revealed significant regional differences in the proportional contribution of environmental microorganisms to the gut microbiota, with individuals in resource-poor habitats (ALK region) exhibiting higher input from soil microbes (8.0672% ± 6.9291%; *p* < 0.05). In conclusion, this study clarifies the ecological mechanism by which soil physicochemical properties regulate the diversity and composition of herbivore gut microbiota through a “soil–plant–food–gut microbiota” cascading pathway, providing important empirical evidence for understanding animal–microbe–environment interactions and adaptive evolution in extreme environments.

## 1. Introduction

The Qinghai–Tibet Plateau is the largest high-altitude plateau globally, serving as both a sensitive and amplifying region for climate change and a crucial natural laboratory for studying the evolution of global biodiversity and species adaptation [1]. Located in the northeastern part of the Qinghai–Tibet Plateau, the Qaidam Basin is one of China’s largest inland basins, with an average elevation of approximately 2800 m. Characterized by an arid and cold climate, low precipitation, and evaporation far exceeding precipitation, the basin commonly experiences soil salinization and desertification. Vegetation is sparse and distributed in patches, making it a typical arid desert ecosystem [2]. Such extreme and diverse environmental conditions not only restrict vegetation growth but also impose survival pressures on wildlife populations inhabiting the area, rendering the Qaidam Basin a significant region for studying ecological adaptation strategies of animals.

The Qaidam subspecies of the goitered gazelle (*Gazella subgutturosaa*; Güldenstädt, 1780) is a predominant large herbivore in the Qaidam Basin and serves as a crucial flagship species for sustaining energy flow and nutrient cycling within the regional ecosystem [3,4,5]. As the only hoofed species with a stable distribution in the core area of the basin, its population dynamics and ecological strategies are essential for the stability of the desert-steppe ecosystem. However, the region faces multiple environmental pressures, including resource scarcity, exacerbated soil salinization, scarce precipitation, and anthropogenic disturbances [6,7,8]. Under such conditions, how goitered gazelles maintain energy acquisition and health through physiological and gut-microbiological mechanisms remains insufficiently investigated.

Gut microbiota can rapidly respond to changes in diet and environmental stress, thereby helping the host maintain homeostasis and ecological adaptability [9,10,11,12]. The composition and diversity of gut microbiota are influenced by multiple factors, including food type, host genetic background, habitat characteristics, and exogenous microbial input [13,14]. Among these, soil and aquatic microbiota constitute the important exogenous microbial sources for herbivores besides food itself: soil represents the planet’s largest microbial reservoir, driving organic matter decomposition, nutrient cycling, and energy flow, and its physicochemical properties—such as organic carbon, nitrogen content, salinity, and cation exchange capacity—directly shape the structure and function of soil microbial communities [15,16]; meanwhile, water serves as a key medium for microbial dispersal and animal ingestion, with aquatic microbiota potentially influencing the colonization and renewal of host gut microbiota [17,18]. However, in the field of terrestrial wildlife conservation biology, the contribution of environmental microorganisms to host gut microbiota has often been overlooked.

The diversity and composition of soil and aquatic microbiota exhibit significant spatial heterogeneity across different regions [19]. Herbivores can ingest environmental microorganisms through geophagy, soil licking, and drinking water—a process that may serve as an important pathway for hosts to modulate their gut microbiota composition [5]. Furthermore, soil physicochemical properties indirectly affect plant community productivity and food availability by altering the diversity and function of soil microbial communities, thereby further influencing host gut microbiota and energy metabolism [20,21]. Therefore, a systematic investigation into the relationships among soil physicochemical properties, soil and aquatic microbial communities, and host gut microbiota is key to understanding the adaptation strategies of herbivores in arid desert regions.

Based on this, the present study focuses on goitered gazelle populations across six typical regions of the Qaidam Basin. We hypothesize that soil physicochemical properties influence environmental conditions, which in turn influence the gut microbial diversity of goitered gazelles and affect the contribution of environmental microorganisms to the gut microbiota. This work aims to analyze the shaping effect of soil physicochemical properties on the gut and soil microbial communities of goitered gazelles, quantify the contribution of environmental microorganisms to gut microbiota, and explore the adaptive strategies and ecological implications of the gut microbiota in Qaidam Basin goitered gazelles. This research will help deepen the understanding of the “host–microbiota–environment” interaction mechanisms in flagship species of arid regions, providing a scientific basis for the targeted conservation and habitat management of flagship species within the Qinghai–Tibet Plateau.

## 2. Materials and Methods

### 2.1. Sample Collection

**Figure 1:**
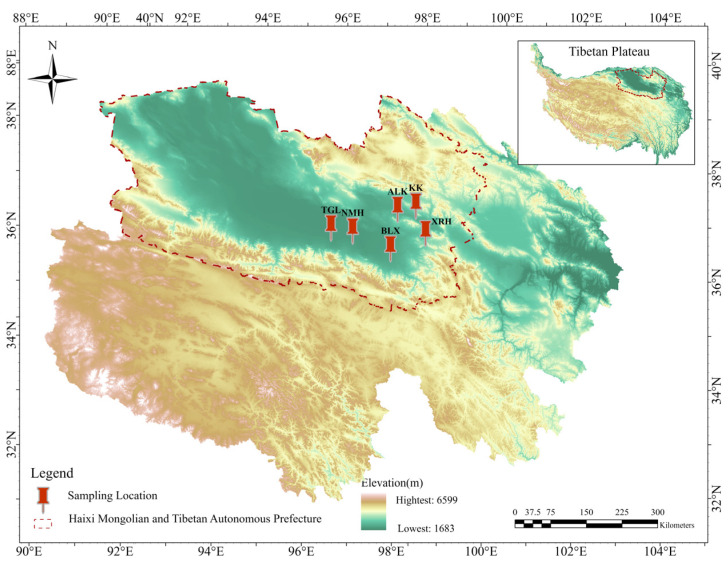
Six sampling locations of goitered gazelles in Qinghai Province, China. Xiariha is abbreviated as XRH; Keke is abbreviated as KK; Aileken is abbreviated as ALK; Balong County is abbreviated as BLX; Nuomuhong is abbreviated as NMH; Tiangeli village is abbreviated as TGL.

The sampling sites for this study were located in Ulan County and Dulan County within the Qaidam Basin. Sampling was conducted from 11 to 20 July 2020. Six regions where goitered gazelles are relatively concentrated and exhibit relatively fixed movement routes were selected as sampling sites (Figure 1). A total of 128 samples (fecal, *n* = 58; soil, *n* = 35; water, *n* = 35) were collected across six regions. The detailed distribution of fecal, soil, and water samples for each region was ALK (10, 5, 5), BLX (8, 4, 7), KKZ (10, 5, 5), NMH (11, 6, 8), TGL (10, 8, 4), and XRH (9, 7, 6), respectively.

When collecting fecal samples, clusters of fresh feces were typically selected. Disposable PE gloves were worn for collection and discarded immediately after use to avoid cross-contamination. During collection, care was taken to minimize contamination from soil, plant stems, leaves, and other impurities. The collected fresh fecal samples were placed in zip-lock bags, labeled and wrapped in aluminum foil, and preserved in liquid nitrogen immediately. All samples were obtained by waiting for animals to defecate naturally, without chasing, startling, or using any drugs on the animals.

To ensure the representativeness of soil samples, surface soil samples were collected within a 200 m radius around each fecal sampling point. Each composite surface soil sample was prepared by mixing equal proportions of five surface soil subsamples collected 50 m apart and passed through a 60-mesh sieve. The samples were then placed into 5 mL cryovials and stored in liquid nitrogen. The latitude and longitude of the soil collection sites were recorded as the same as those of the corresponding fecal samples.

For aquatic microbial samples, approximately 1 L of water was filtered through a filter membrane (biosharp, Beijing, China). The filter membrane was wrapped in aluminum foil, labeled, and preserved in liquid nitrogen. Water samples were collected from water sources located within 1 km of the fecal sampling points; the water collection sites were recorded as the same as those of the corresponding fecal samples.

### 2.2. DNA Extraction, Amplification, and Sequencing of Feces, Soil, and Water Microbiota

To ensure that all data are amenable to unified analysis, identical procedures were applied to all samples. Total genomic DNA was extracted from fecal samples using the E.Z.N.A.^®^ Soil DNA Kit (Omega Bio-tek, Norcross, GA, USA) following the manufacturer’s instructions. DNA quality was verified by 1% agarose gel electrophoresis. The extracted DNA was assessed for concentration, purity, and potential contamination using an ultra-micro spectrophotometer (NanoDrop2000, Thermo Fisher Scientific, Waltham, MA, USA). Qualified samples were then subjected to electrophoresis on a 1% agarose gel at 5 V/cm for 20 min to evaluate DNA integrity. Samples that passed quality assessment were used for subsequent experiments.

PCR amplification was performed using universal primers 338F and 806R [22] targeting the V3-V4 region. The PCR reaction system had a total volume of 20 μL. Amplification was conducted using an ABI GeneAmp^®^ 9700 PCR instrument (Thermo Fisher Scientific, MA, USA). The PCR products were checked by electrophoresis on a 2% agarose gel. Target bands were recovered and purified using the AxyPrep DNA Gel Extraction Kit (Corning Incorporated, Corning, NY, USA).

Following the instructions of the NEXTflex^®^ Rapid DNA-Seq Kit (PerkinElmer, Waltham, MA, USA) and standardized library construction procedures, a sequencing library was constructed for the gel-purified 16S rRNA gene V3-V4 fragments. Qualified sequencing libraries were subjected to PE300 (paired-end) sequencing on the Illumina MiSeq platform (Illumina, San Diego, CA, USA). The corresponding sequencing library construction and sequencing were undertaken and completed by Majorbio Bio-Pharm Technology Co., Ltd. (Shanghai, China).

### 2.3. Quality Control of Microbial Sequencing Data

ASV (Amplicon Sequence Variant) sequences and their relative abundances were obtained using the DADA2 (v 1.30.0) [23] (Divisive Amplicon Denoising Algorithm 2) denoising approach. In this study, the upstream sequence analysis of the 16S rRNA gene V3-V4 region was performed within QIIME2 [22].

The fastx_barcode_splitter.pl script from the FastX-toolkit (v 0.0.14) was used to demultiplex the barcodes from the raw sequencing data [24]. Denoising was conducted using DADA2 (https://benjjneb.github.io/dada2/tutorial.html, accessed on 27 December 2025), with sequence trimming performed by the fastp software (v 0.24.0) [25]. To enhance the sensitivity of ASV identification, the pseudo-pooling method was employed during the denoising process. This yielded the raw ASV sequences and an ASV abundance table for all samples.

Taxonomic annotation of the raw ASV sequences was performed using the feature-classifier classify-sklearn tool in QIIME2 (main parameters: --*p*-confidence 0.8, --*p*-read-orientation) [26,27], with the confidence threshold set to 0.8. The taxa filter-table tool in QIIME2 was then used to filter out entries annotated as mitochondria, chloroplast, or Archaea based on the ASV taxonomic assignment results. Based on the ASV abundance table, ASVs with a relative abundance below one per million and those appearing in fewer than five samples were filtered out. The quality-controlled ASV sequences and abundance table were used for all subsequent analyses.

### 2.4. Normalization and Analysis of ASVs

The phylogenetic tree of ASV sequences was constructed using the QIIME2 phylogeny align-to-tree-mafft-fasttree pipeline with default parameters [28]. For standardization, rarefaction was applied to the ASV abundance table using the feature-table rarefy function in QIIME2, with a rarefaction depth set to 43,470 sequences, corresponding to the sample with the lowest sequencing depth. In all subsequent analyses, unless otherwise specified, the rarefied ASV abundance table and corresponding ASV sequences were used. An exception was made for the FEAST source-tracking analysis [29], which requires the original non-rarefied ASV abundance table.

Based on the rarefied ASV table and the corresponding phylogenetic tree, α-diversity indices for each sample were calculated using the microeco package (v 1.10.0) [30]. These included Observed richness, Shannon index, and Faith’s phylogenetic diversity (PD index), evaluating microbial communities from the perspectives of abundance, evenness, and phylogenetic diversity, respectively. The Kruskal–Wallis rank sum test from the R stats package (v 4.4.1) [31] was employed to assess significant differences in α-diversity indices among gut, soil, and aquatic microbiota, with a significance threshold of *p* < 0.05.

Based on ASV relative abundances, the Bray–Curtis distance was calculated for fecal and soil samples separately to quantify differences in microbial community composition between samples. This distance metric served as the primary response variable in subsequent analyses associated with soil physicochemical properties. Permutational Multivariate Analysis of Variance (PERMANOVA) from the vegan package (v 2.6-8) [32] was performed to test for significant differences in β-diversity (microbial community composition) among gut, soil, and aquatic microbiota, with *p* < 0.05 considered statistically significant. Results were visualized using the ggplot2 package (v 3.5.1) in R [33].

### 2.5. Source Tracing Analysis

Source tracking analysis of gut microbiota, also known as microbial source tracking, is primarily used to determine the origins of microbial species within the gut. Fast Expectation-maximization for microbial Source Tracking (FEAST) [29] is a method for microbial source tracking that enables more accurate and rapid estimation of the proportional contributions from various sources.

Using the R package FEAST (version new beta, https://github.com/cozygene/FEAST, accessed on 29 December 2025), with the number of EM iterations set to 10,000,000, a source tracking analysis was performed on the gut microbiota of goitered gazelles (main parameters: COVERAGE = 26,685, EM_iterations = 10,000,000). In this process, each fecal sample was defined as a “sink” for tracking microbial origins, while the corresponding soil and water samples collected from the same location were defined as “sources,” representing potential origins of the gazelles’ gut microbiota. The Kruskal–Wallis test for the source tracking results was performed using SPSS 27.

### 2.6. Determination and Data Normalization of Soil Physicochemical Properties

The measurement of the 11 soil indicators followed the earlier described methods [34] described in the book *Soil Physical and Chemical Analysis & Description of Soil Profiles* edited by Liu Guangsong in 1996. pH was measured using the potentiometric method; Soil Organic Carbon (SOC) was determined by the potassium dichromate oxidation heating method; Total Nitrogen (TN) was measured using the Semimicro-Kjeldahl Method; Total Phosphorus (TP) was analyzed via hydrofluoric acid-perchloric acid digestion followed by the molybdenum antimony anti-colorimetric method; Total Potassium (TK) was determined by hydrofluoric acid-perchloric acid digestion and flame photometry method; Exchangeable Calcium (ExCa) and Exchangeable Magnesium (ExMg) were measured using the ammonium acetate exchange-atomic absorption spectrometry method; Exchangeable Sodium (ExNa) was analyzed by ammonium acetate exchange-flame photometry; Cation Exchange Capacity (CEC) was determined using the ammonium acetate exchange method; Particle Density (PD) was measured via the pycnometer method; and Total Salinity (TS) was determined using the gravimetric method.

Given that the number of soil physicochemical property samples was typically lower than that of microbial samples (generally 3–6 soil samples per region, compared to 8–10 corresponding fecal or soil 16S samples), a “regional mean imputation” approach was adopted to ensure comparability between the two datasets at the sample scale. Specifically, the mean value of each soil physicochemical property within a given sampling region was assigned to all 16S rRNA samples from that region, thereby constructing a fully aligned environmental variable matrix.

### 2.7. Correlation Analysis and Screening of α-Diversity and Soil Physicochemical Properties

Prior to correlation analysis, the distribution of the data was tested using the shapiro.test function from the R stats package [31]. The results indicated that the soil physicochemical property data did not follow a normal distribution. Therefore, all correlation tests in this study were performed using Spearman. To preliminarily screen for soil physicochemical properties significantly associated with α-diversity, pairwise correlation analysis between environmental variables and diversity indices was conducted using Spearman’s rank correlation coefficient. Given the simultaneous comparison of multiple environmental variables, the False Discovery Rate (FDR) method was applied to correct the *p*-values for multiple comparisons. Correction was performed separately for each diversity index group to control the false positive rate under each index. For each index, adjusted *p*-values < 0.05 were considered indicative of a significant correlation.

Building on this, and to avoid including highly correlated soil physicochemical properties in subsequent linear models—thereby mitigating the impact of multicollinearity on regression coefficients and significance tests—cluster analysis based on Spearman correlation was performed on the environmental variables significantly correlated with the α-diversity indices. This analysis was conducted using the varclus function from the R Hmisc package (v 5.2-0) [35] (main parameter: method = “spearman”). Variables exhibiting high correlation were filtered using the absolute value of the Spearman correlation coefficient (|*ρ*| ≥ 0.65) as the threshold. During variable selection, a principle was followed to prioritize retaining the factor that could maximally explain other correlated variables.

### 2.8. Construction and Selection of Multivariate Linear Models for α-Diversity

Following significance screening and collinearity treatment, ordinary least squares multiple linear regression models were constructed to quantitatively assess the explanatory power of soil physicochemical properties on α-diversity. The standardized Observed, Shannon, and PD indices served as the response variables, respectively, with the filtered soil physicochemical properties as the independent variables. For each response variable, a full model containing all candidate independent variables was first fitted. To identify the most parsimonious model with good explanatory power, the dredge function from the MuMIn package (v 1.48.4) [36] was then used to perform exhaustive subset model selection and Akaike Information Criterion (AIC) comparison based on the given full model. Candidate models with lower ΔAIC, higher explanatory power (R^2^), and significant parameters were selected. For the optimal or sub-optimal models identified, the model R^2^, the standardized regression coefficients of each independent variable, and their significance levels were extracted. The absolute value of each standardized coefficient was used as an indicator of the relative importance of the corresponding predictor variable.

### 2.9. Mantel Correlation Analysis Between β-Diversity and Soil Physicochemical Properties

For β-diversity analysis, the Bray–Curtis distance matrix served as the response variable representing microbial community differences. A corresponding Euclidean distance matrix was constructed for each soil physicochemical property, forming a list of environmental distance matrices. To systematically assess the correlation between community β-diversity and each environmental distance, Mantel tests [37] were performed for all “community distance × environmental distance” combinations within the fecal and soil datasets separately. Each Mantel test estimated the significance of the correlation coefficient based on 999 random permutations.

In both fecal and soil samples, soil physicochemical variables with *p* < 0.01 and a Mantel correlation coefficient r > 0.25 were considered significant. Since the Spearman correlation coefficients among most Mantel-significant factors were generally ≥0.65, indicating substantial multicollinearity, subsequent MRM [38] models needed to select a single “proxy variable” from each highly collinear cluster to avoid model instability. Following this principle, pH, TP, and ExMg were included in the MRM model for fecal microbiota, while ExMg and ExNa were included in the model for soil microbiota. These variables were selected from the clusters of Mantel-significant variables as the most representative within their respective clusters.

### 2.10. Multivariate Regression Analysis of β-Diversity Using MRM

To quantify the combined explanatory power of multiple soil physicochemical distance matrices on the variation in microbial community β-diversity, the MRM method from the ecodist package (v 2.1.3) was employed for multivariate regression analysis between the Bray–Curtis distance and the significant environmental distance matrices. For fecal samples, the Bray–Curtis distance matrix calculated from the rarefied ASV table served as the response variable, with the standardized Euclidean distance matrices of soil pH, total phosphorus (TP), and exchangeable magnesium (ExMg) included as independent variables after standardization (stdize). For soil samples, the Bray–Curtis distance of the soil microbial community was the response variable, and the standardized Euclidean distance matrices of exchangeable magnesium (ExMg) and exchangeable sodium (ExNa) were the independent variables. All MRM models were run on rank-transformed distances (mrank = TRUE) to mitigate the influence of extreme distance values. The significance of model parameters and the overall model was estimated through 999 random permutations.

## 3. Results

### 3.1. Source Tracking

In the soil source-tracking analysis results (Table 1), the ALK region showed the highest contribution (8.07%), while BLX showed the lowest (0.12%), with a significant difference between the two groups (*p* < 0.01). For the aquatic microbial source tracking, XRH had the highest contribution (7.64%), and BLX had the lowest (0.15%), also with a significant inter-group difference (*p* < 0.01). Regarding the combined results of environmental microbial source tracking, ALK was highest (10.40%) and BLX was lowest (0.27%). Based on the Kruskal–Wallis test results, the source-tracking contributions of both soil and aquatic microorganisms showed significant differences among the six groups (*p* < 0.05).

### 3.2. Soil Physicochemical Properties

Among the 11 soil physicochemical indicators, soil organic carbon, total nitrogen, exchangeable magnesium, exchangeable sodium, exchangeable calcium, CEC, and total salinity showed significant differences across regions (Kruskal–Wallis, *p* < 0.05). NMH had the highest values for soil organic carbon, total nitrogen, exchangeable magnesium, particle density, and total salinity (Table 2). ALK had the lowest soil organic carbon, total nitrogen, and total salinity. XRH and KKZ exhibited the highest exchangeable calcium content. Soil pH across all regions ranged between 7.4 and 8.6, indicating weakly alkaline conditions with little variation among regions.

### 3.3. Correlations Between Soil Physicochemical Properties and the α-Diversity of Gut and Soil Microbiota in Goitered Gazelles

The Observed index of gut microbiota was influenced by multiple environmental variables. It showed significant positive correlations with TP (rho = 0.419, *p* < 0.01) and ExCa (rho = 0.401, *p* < 0.01), while exhibiting significant negative correlations with SOC, TN, TS, and ExMg (rho approximately −0.35 to −0.38, *p* < 0.02). The correlation pattern for the PD index of gut microbiota was highly consistent with that of the Observed index. The Shannon index of gut microbiota was significantly correlated with only three soil physicochemical properties: it showed a significant negative correlation with pH (rho = −0.519, *p* < 0.001) and significant positive correlations with TP and PD (both rho ≈ 0.41, *p* < 0.01). This indicates that microbial community evenness is more sensitive to soil pH and total phosphorus content.

In contrast, the α-diversity of soil samples exhibited a more distinct pattern. The soil Observed index showed the strongest correlations with SOC, TS, and ExMg (rho = −0.61, *p* < 0.001) and a significant negative correlation with TN. Simultaneously, ExCa (rho = 0.49, *p* < 0.01) and TK (rho = 0.50, *p* < 0.01) had significant positive effects on richness. The correlation pattern for the soil PD index was consistent with that of the soil Observed index. The soil Shannon index had the highest number of significantly correlated variables: SOC, TS, ExMg, TN, and ExNa had negative influences, while ExCa (rho = 0.63, *p* < 0.001), TK (rho = 0.53, *p* < 0.01), and TP (rho = 0.38, *p* < 0.05) were significantly positively correlated with the soil Shannon index.

### 3.4. Screening of Linear Models and Optimal Model Results for the Observed, Shannon and PD Indexes of Gut Microbiota in Goitered Gazelles

Based on the all-subsets regression analysis of all candidate environmental variables (Table 3), model comparison results indicated that the two-variable model containing SOC and TP held an absolute advantage. It had the lowest corrected AIC (AICc = 150.368) and a model weight as high as 0.989, far exceeding other candidate models. This suggests overwhelming relative support for this model among all comparable models. Although the univariate model containing only SOC and the one containing only TP were included in the candidate set, their AICc values were significantly higher than that of the optimal model (ΔAICc > 9), with weights of only 0.008 and 0.003, respectively. This indicates that these univariate models have insufficient explanatory power for the data when predicting the richness (Observed index) of gut microbiota in goitered gazelles.

According to the general criterion for model selection (models with ΔAICc < 2 can be considered equally competitive), only the SOC + TP model met the standard in this study and was therefore identified as the optimal predictive model for the Observed index of gut microbiota in goitered gazelles.

The parameter estimation results of the optimal model further revealed that SOC and TP exerted opposing effects on gut microbial species richness (Table 4). The model’s explanatory power (deviance R^2^) was 0.3154, and the overall model was significant (*p* = 2.985 × 10^−5^), indicating that the gradient of soil physicochemical properties could explain approximately 31.5% of the variation in richness. In terms of standardized coefficients, the regression coefficient for SOC was −0.44, with a 95% confidence interval of [−0.66, −0.21], reaching a highly significant level (*p* = 0.000257). This shows that as soil organic carbon content increases, the Observed index of gut microbiota in goitered gazelles exhibits a significant decreasing trend. The standardized coefficient for TP was 0.40, with a confidence interval of [0.17, 0.62], also highly significant (*p* = 0.00081), indicating that increased total phosphorus content can significantly promote an increase in species richness.

Overall, the model screening and parameter estimation results consistently demonstrate that SOC (negative effect) and TP (positive effect) constitute the most critical soil physicochemical properties driving species richness in the fecal microbiota of goitered gazelles. They are not only statistically significant but also exhibit strong and stable model support in the all-subsets comparison.

Based on all-subsets regression of all candidate soil physicochemical properties, three candidate models with ΔAICc less than 2 were identified, indicating these models possess similar and relatively strong statistical support in explaining the variation in the Shannon index (Table 5). The model with the lowest AICc, composed of pH and TP (Model 1), had the highest model weight (0.377), making it the optimal explanatory model for the current data. Additionally, the three-variable model containing pH, TP, and PD (weight 0.286) and the two-variable model composed of TP and PD (weight 0.189) also demonstrated strong competitiveness. The AICc values of the remaining models were significantly higher, and their weights considerably lower (<0.11), indicating limited explanatory power for the Shannon index. According to information-theoretic criteria, models with ΔAICc < 2 are generally considered statistically equivalent; therefore, the three aforementioned models were used for subsequent parameter estimation and comparison in this study.

Linear regression results for the three candidate models revealed that the variation in the Shannon index of goitered gazelle gut microbiota is primarily driven collectively by pH, TP, and PD, although the direction of effect and significance of each variable differed among models (Table 6). In Model 1 (pH + TP), pH had a significant negative effect on the Shannon index (standardized coefficient = −0.41, 95% CI = [−0.65, −0.18], *p* < 0.001), indicating that as soil pH increases, gut microbial evenness decreases. TP showed a significant positive effect (coefficient = 0.25, *p* = 0.0338), suggesting that increased total phosphorus may promote gut microbial evenness. The overall explanatory power (R^2^) of this model was 0.2539, and the model was globally significant (*p* = 3.18 × 10^−4^).

Model 2 (pH + TP + PD) showed a slight improvement in explanatory power (R^2^ = 0.2773). However, the estimated effects of both pH and TP did not reach significance (*p* > 0.05), and their confidence intervals crossed zero. Only PD showed a significant positive correlation (coefficient = 0.36, *p* = 0.0136). This suggests that although the three-variable model fits the data slightly better overall, the independent contributions of pH and TP within this model are not stable, possibly due to variable interaction or collinearity.

In Model 3 (TP + PD), both TP and PD had significant positive effects on the Shannon index (TP coefficient = 0.43, *p* < 0.01; PD coefficient = 0.48, *p* < 0.001). The model’s explanatory power was 0.236, slightly lower than Models 1 and 2. This model indicates that when pH is excluded, TP and PD are two key factors that can stably enhance Shannon diversity.

Integrating AICc, model weights, and parameter significance, the results show that the variation in the Shannon index is influenced jointly by gradients of three main soil physicochemical properties: the negative effect of pH, and the positive effects of TP and PD. Among these, the combination of pH and TP demonstrated the most robustness in terms of both significance and model parsimony, while the effect of PD became more prominent after removing pH. This illustrates that the evenness of gut microbiota in goitered gazelles is regulated by the combined effects of soil pH, total phosphorus, and particle density, exhibiting an ecological response structure driven by multiple factors.

Following all-subsets regression of all candidate environmental variables, model selection results indicated that the combined model of SOC and TP held an absolute advantage in explaining the PD index of gut microbiota in goitered gazelles (Table 7). This model had the lowest AICc among all candidates (AICc = 153.983), with a model weight as high as 0.943, meaning it received almost all the support under the current data conditions. The AICc values of all other models were ≥5 higher than this optimal model, and their weights decreased significantly (≤0.052), indicating their insufficient explanatory power for the PD index. Therefore, only the two-variable model containing SOC and TP was carried forward for subsequent analysis.

Linear regression results based on this optimal candidate model revealed that both SOC and TP had significant, yet opposing, effects on the PD index of goitered gazelle gut microbiota (Table 8). The standardized coefficient for SOC was −0.436 (95% CI = [−0.67, −0.16], *p* < 0.001), indicating that higher soil organic carbon content is associated with lower phylogenetic diversity of gut microbiota in goitered gazelles. The standardized coefficient for TP was 0.332 (95% CI = [0.10, 0.57], *p* < 0.01), suggesting that higher total phosphorus levels in the habitat soil are associated with higher phylogenetic diversity of gut microbiota. The overall model was significant (*p* = 1.66 × 10^−4^) and explained 27.13% of the variation in the gut microbial PD index, demonstrating that SOC and TP are key gradients of soil physicochemical properties influencing the phylogenetic diversity of gut microbiota in goitered gazelles.

### 3.5. Mantel Correlation Analysis Between Soil Physicochemical Properties and β-Diversity of Gut and Soil Microbiota in Goitered Gazelles

**Figure 2:**
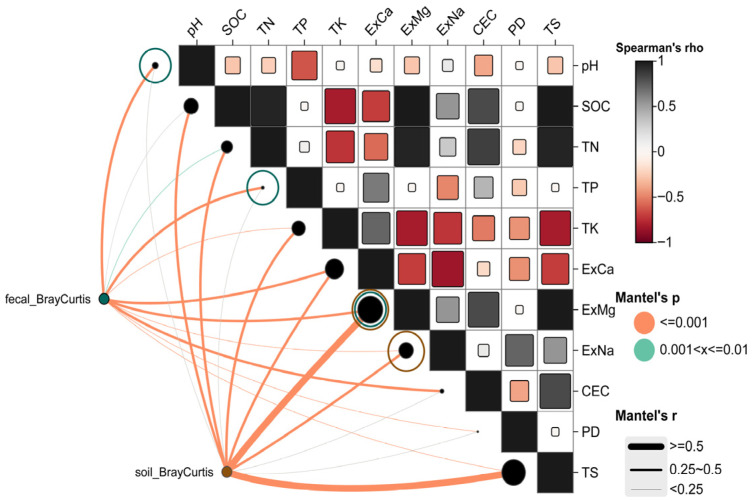
Mantel correlation analysis between soil physicochemical properties and Bray–Curtis distances of goitered gazelles’ gut microbiota and environmental soil microbiota separately.

For the soil microbial community, Bray–Curtis distances showed significant positive correlations with ExMg (r = 0.621), TS (r = 0.599), SOC (r = 0.472), TK (r = 0.392), ExNa (r = 0.372), ExCa (r = 0.370), and TN (r = 0.353) (*p* ≤ 0.001) (Mantel r values primarily ranged between 0.25 and 0.50 or >0.50). These results (Figure 2) indicate that the compositional differences in soil microbial communities are primarily driven by soil mineral elements (ExMg, ExCa, ExNa, TK) and carbon-nitrogen content (SOC, TN). In contrast, the Mantel correlations for pH, TP, CEC, and PD were not significant (*p* > 0.05, represented by gray lines), suggesting their limited influence on soil microbial β-diversity.

For the gut microbial community (Figure 2), Bray–Curtis distances showed significant relationships with pH (r = 0.388), ExCa (r = 0.347), TP (r = 0.323), ExMg (r = 0.261), CEC (r = 0.281), ExNa (r = 0.247), TS (r = 0.248), TK (r = 0.201), TN (r = 0.187), and PD (r = 0.214) (*p* ≤ 0.001 or 0.01 < *p* ≤ 0.01). Compared to soil microbiota, the Mantel r values for gut microbiota were generally in a moderate range (approximately 0.14–0.39). This suggests that while the β-diversity of gut microbiota is significantly correlated with gradients of soil physicochemical properties, the strength of association is less pronounced than for soil microbiota. This difference may reflect the integrated influence of host diet, intestinal environment, and environmental microbial input on the gut microbial community.

Given the high collinearity among the significant variables identified by the Mantel test, with Spearman correlation coefficients generally ≥0.65, a single “proxy variable” was selected from each highly correlated cluster for subsequent MRM models to avoid the impact of multicollinearity on model stability. Consequently, pH, TP, and ExMg were included in the MRM model for gut microbiota, while ExMg and ExNa were included in the model for soil microbiota.

### 3.6. MRM Analysis on β-Diversity (Bray–Curtis Distance) of Gut and Soil Microbiota in Different Geographic Populations of Goitered Gazelles

For the gut microbial community, the optimal model composed of pH, TP, and ExMg explained 24.03% of the community variation (R^2^ = 0.2403, *p* = 0.001), indicating that gut microbial β-diversity is jointly influenced by multiple soil physicochemical factors. Both pH (coef = 0.3083, *p* = 0.001) and TP (coef = 0.227, *p* = 0.003) had significant positive prediction on the Bray–Curtis distance, this indicates that greater regional differences in soil pH or total phosphorus content are associated with greater dissimilarity between gut microbial communities (Table 9). Although ExMg did not reach the significance threshold (coef = 0.0944, *p* = 0.0511), exchangeable magnesium may still contribute to the variation in gut β-diversity to some extent. By pairing the environmental distances of significant predictor variables with the gut microbial Bray–Curtis distances (Table 9), it can be observed that greater differences in soil pH, TP, and ExMg between regions—indicating stronger soil heterogeneity—are associated with greater differences in gut microbial communities among goitered gazelles.

For the soil microbial community, the optimal model composed of ExMg and ExNa explained 35.07% of the community variation (R^2^ = 0.3507, *p* = 0.001). ExMg had a significant positive prediction on soil microbial β-diversity (coef = 0.5275, *p* = 0.001) (Table 9), representing the primary driving factor in this model. While ExNa also showed a positive prediction (coef = 0.0966), it was not significant (*p* = 0.2603) (Table 9), indicating a weaker contribution. Overall, ExMg plays a central role in shaping soil microbial β-diversity.

Integrating the MRM results, it is evident that although gut microbial β-diversity is co-influenced by multiple soil physicochemical gradients (pH, TP, and ExMg), its R^2^ is significantly lower than that of the soil microbial community. This reflects the complex regulation of gut microbiota by host physiology, dietary composition, and environmental microbial input. In contrast, soil microbial β-diversity demonstrates higher environmental sensitivity, showing a stronger response specifically to mineral elements.

## 4. Discussion

### 4.1. Effects of Soil TP and SOC on the α-Diversity of Gut Microbiota

The results of this study indicate that the most important soil characteristics influencing gut microbial diversity are total phosphorus (TP) and organic carbon (SOC). It is generally accepted that higher gut microbial diversity, as observed in relation to environmental conditions, is associated with greater functional redundancy and better host health in host–microbe systems [39]. In this study, soil TP showed a significant positive correlation with the α-diversity of gut microbiota in goitered gazelles. Phosphorus is typically considered a crucial nutrient element affecting vegetation productivity and plant growth [40], and plant diversity is significantly correlated with soil total phosphorus concentration [41]. In the arid and nutrient-limited ecosystem of the Qaidam Basin, phosphorus enrichment in soil can increase the number of plant species and improve the abundance and stability of dominant species [42,43], thereby providing goitered gazelles with more diverse food choices. Furthermore, soil TP can alter the nutritional composition within plant tissues by regulating plant nutritional quality [44]. As typical herbivores, the gut microbiota of goitered gazelles is highly susceptible to dietary influences [45]. Therefore, when goitered gazelles forage in nutrient-sufficient and vegetation-rich areas, the diversification of food types and the improvement of plant nutritional quality jointly provide richer nutritional sources for their gut microbiota, favoring the existence of a wider range of microbial taxa [46], ultimately collectively enhancing gut microbial α-diversity.

In contrast to the positive correlation between soil TP and gut microbiota, soil organic carbon (SOC) showed a significant negative correlation with the α-diversity of gut microbiota in goitered gazelles. Soil organic carbon is a key indicator reflecting the soil physicochemical environment. In arid, saline-alkali, and resource-limited ecosystems like the Qaidam Basin, an increase in SOC is often accompanied by the dominance of a few stress-tolerant plant taxa [47], and plant species diversity indices are negatively correlated with SOC [48]. Soils with high SOC also affect the nutritional composition of plant tissues [42], causing plants to exhibit conservative traits such as higher structural carbon content and higher C:N ratios, thereby reducing the nutritional quality available to herbivores [49]. The results of this study also show that soil SOC was significantly negatively correlated with soil microbial α-diversity (Observed and PD indices). This may be because under high SOC conditions, certain microbial taxa become dominant within the community, subsequently affecting the distribution and coexistence of other microbial groups and leading to reduced soil microbial diversity [50,51]. Changes in soil microbial community structure not only affect the decomposition of soil organic matter and nutrient mineralization processes but can also regulate plant nutrient uptake and growth status through rhizosphere microbe-plant interactions [52]. In summary, high SOC may indirectly limit the food resources available to goitered gazelles by reducing plant species diversity, altering plant nutritional composition, and interacting with soil microbial community structure, thereby affecting their gut microbial α-diversity [53,54].

### 4.2. Environmental Heterogeneity Drives Spatial Differentiation of Gut Microbiota

The results from the MRM predictive model indicate that soil pH, total phosphorus (TP), and exchangeable magnesium (ExMg) are the primary predictors of variation in the β-diversity of gut microbiota in goitered gazelles. The synergistic effects of soil nutrients and pH are important factors for plant growth, including species distribution and adaptation [55]. We propose that the spatial differentiation of gut microbiota may be linked to the uneven distribution of plant resources [56]. Within the ecologically extreme context of the Qaidam Basin, characterized by aridity and high salinity-alkalinity, vegetation distribution and nutritional composition are typically constrained by multiple environmental factors rather than a single independent one. Soil pH and ExMg, serving as key physicochemical indicators reflecting the degree of salinization and ionic composition, exert selective pressures on plant tolerance across larger spatial scales, thereby influencing the distribution patterns of different vegetation types [57,58]. In contrast, TP, as a key limiting nutrient element in the Qaidam Basin ecosystem, is closely related to plant biomass accumulation and growth performance [59]. The heterogeneity of soil physicochemical properties collectively influences the species and nutritional content of plants foraged by goitered gazelles across different habitats in the Qaidam Basin. By consuming these plants, the heterogeneity of food resources imposes selective pressures on their gut microbiota, consequently affecting its β-diversity [60,61]. Furthermore, environmental pH and mineral ions themselves are significant physical stress factors. Under field conditions, goitered gazelles are directly exposed to these factors through drinking and foraging, which can alter the osmotic conditions and acid response systems within their intestinal environment, thereby exerting direct selective pressure on the microbial community [19,62]. Specifically, pH and mineral ions influence the abundance of certain microbial taxa and the production of health-related metabolites like short-chain fatty acids (SCFAs), thereby reshaping the gut microbiota and further intensifying the differentiation of gut microbial composition between different habitats [63].

### 4.3. Habitat Characteristics Influence the Source-Tracking Contribution to Gut Microbiota

In this study, source-tracking analysis revealed that goitered gazelles in the ALK region had the highest proportion of gut microbiota derived from soil, while the BLX region had the lowest. The soil physicochemical properties in the ALK region are generally poor [2,64], with the lowest values for indicators such as SOC, TN, TP, and CEC, indicating limited plant growth conditions and relatively scarce food resources in this area. Under these circumstances, the variety of plants available for goitered gazelles to forage is restricted, leading to reduced diversity and stability of their gut microbial communities. Furthermore, more frequent contact with and ingestion of soil and water during foraging and drinking increases exposure to environmental microorganisms, which may serve to supplement the gut microbial community and help maintain its homeostatic balance. This aligns with our previous finding that goitered gazelle populations in resource-poor areas of the Qaidam Basin have a higher proportional contribution from soil microbiota [5]. Moreover, this is confirmed by possible symbiotic relationships between different groups of organisms, which are noted with high frequency in ecosystems [65,66].

In contrast, goitered gazelles in the BLX region showed the lowest source-tracking proportion for gut microbiota. During sampling, we observed that the BLX area was dominated by dense tall shrubs with no herbaceous vegetation in the understory, and the installation of fencing effectively separated the gazelles’ activity range from surrounding farmlands cultivated by herders. These habitat features likely limited the opportunities for goitered gazelles to come into contact with soil and farmland environmental microbes, resulting in a relatively singular composition of their gut microbial sources and a lower source-tracking proportion.

Conversely, the NMH region showed the highest salinity and nutrient levels, yet a low proportion from environmental microbes. We suppose that the “Environmental Filtering” effect is influenced not only by microbial abundance in the soil but also by specific physicochemical stresses (e.g., high salinity in NMH) and environmental conditions [67,68]. The intermediate soil conditions observed in KKZ and XRH are consistent with this gradient, where moderate resource availability could be associated with a more balanced microbial contribution [69,70].

Overall, differences in soil physicochemical properties and habitat structure among regions appear to influence the source composition of gut microbiota in goitered gazelles, likely via effects on vegetation, food resources, and exposure to environmental microbes. This is consistent with our hypothesis that soil physicochemical properties influence the gut microbiota of goitered gazelles.

## 5. Conclusions

This study elucidates the critical links between soil physicochemical properties and the gut microbiota of goitered gazelles in the hyper-arid Qaidam Basin. Soil total phosphorus (TP) showed a significant positive correlation with the α-diversity of gut microbiota, whereas soil organic carbon (SOC) exhibited a significant negative correlation. Concurrently, soil pH, TP, and exchangeable magnesium (ExMg) were closely associated with the spatial differentiation of gut microbial communities. These soil physicochemical factors collectively influence the diversity of gut microbiota in goitered gazelles by affecting vegetation conditions across different habitats. Furthermore, the proportional contribution of soil microorganisms to the gut microbiota of goitered gazelles varies significantly under different habitat conditions, being higher in environments with limited resources and lower in areas with moderate habitat conditions and shrub-dominated vegetation. This study highlights how changes in soil quality and habitat conditions—often influenced by human activities—can affect the health and survival of wildlife in arid regions.

In summary, our findings establish that soil physicochemical properties influence herbivore gut microbiota in extreme environments through a multi-pathway framework: directly by modifying the soil microbiota and indirectly by governing the availability and nutritional value of forage. This research underscores the importance of an integrated “host–microbiota–environment” perspective for understanding ecological adaptation and informs conservation strategies for flagship species in vulnerable arid ecosystems under global change. More importantly, these findings underscore the importance of sustainable land use and environmental management for maintaining ecosystem stability and the long-term coexistence between humans and wildlife in arid regions.

## Figures and Tables

**Table 1:** Results of source tracking analysis in different regions based on FEAST analysis.

Region	Soil Source Tracking Results/%	Water Source Tracking Results/%	Unknown Source/%
KKZ	4.3580 ± 7.8780	1.1097 ± 1.5500	94.5323
XRH	1.3047 ± 4.3300	7.6410 ± 13.7100	91.0543
ALK	8.0672 ± 6.9291	2.3312 ± 5.8901	89.6016
TGL	2.6487 ± 5.2100	0.1802 ± 0.2300	97.1712
NMH	1.3961 ± 1.7612	0.3347 ± 0.5934	98.2692
BLX	0.1207 ± 0.0400	0.1466 ± 0.1476	99.7326

Note: The values in the table are expressed as percentages.

**Table 2:** The mean values of the 11 soil physicochemical properties across the six regions.

Region/Unit	pH	SOC (g/kg)	TN (g/kg)	TP (g/kg)	TK (g/kg)	ExCa (1/2Ca^2+^, cmol/kg)	ExMg (1/2Mg^2+^, cmol/kg)	ExNa (cmol/kg)	CEC (cmol/kg)	PD (g/cm^3^)	TS (%)
KKZ	8.1340 ± 0.3142	3.0088 ± 1.9313	0.1759 ± 0.1921	0.6400 ± 0.0563	16.5990 ± 1.1946	5.0370 ± 0.9183	0.4822 ± 0.2106	0.2913 ± 0.1479	2.5125 ± 0.8904	2.6664 ± 0.0314	0.9234 ± 0.9169
XRH	8.2900 ± 0.0837	4.1438 ± 1.4232	0.3735 ± 0.1273	0.7068 ± 0.0608	15.6946 ± 0.7162	6.2686 ± 0.5861	0.5264 ± 0.1351	0.2795 ± 0.0477	4.3215 ± 0.5831	2.5667 ± 0.1180	0.9807 ± 0.6121
ALK	8.3440 ± 0.2344	1.2756 ± 0.8703	0.0800 ± 0.0607	0.3901 ± 0.1703	16.9182 ± 2.5178	3.5772 ± 1.1892	0.2708 ± 0.1138	0.3000 ± 0.3428	1.8090 ± 1.2851	2.6648 ± 0.0135	0.8353 ± 1.2150
TGL	8.3100 ± 0.0787	10.4186 ± 2.1222	0.4098 ± 0.1742	0.5576 ± 0.1657	12.9965 ± 1.4975	2.1639 ± 0.8095	2.4435 ± 1.6479	0.9384 ± 0.5267	3.1658 ± 0.8084	2.6800 ± 0.1096	12.7133 ± 4.3566
NMH	8.2400 ± 0.3199	27.4981 ± 42.0476	0.9021 ± 1.2635	0.5845 ± 0.1868	15.5384 ± 5.4536	2.2755 ± 0.4684	3.3206 ± 2.2903	0.8636 ± 0.2049	4.6961 ± 7.9254	2.6310 ± 0.1219	19.3143 ± 15.6460
BLX	8.25 ± 0.1142	4.5658 ± 4.5658	0.1099 ± 0.1099	0.1220 ± 0.1220	3.6733 ± 3.6733	1.4149 ± 1.4149	1.5256 ± 1.5256	0.9918 ± 0.9918	1.4442 ± 1.4442	0.0369 ± 0.0369	14.3887 ± 14.3887

Note: Soil Organic Carbon is abbreviated as SOC; Total Nitrogen is abbreviated as TN; Total Phosphorus is abbreviated as TP; Total Potassium is abbreviated as TK; Exchangeable Calcium is abbreviated as ExCa; Exchangeable Magnesium is abbreviated as ExMg; Exchangeable Sodium is abbreviated as ExNa; Cation Exchange Capacity is abbreviated as CEC; Particle Density is abbreviated as PD; Total Salinity is abbreviated as TS.

**Table 3:** Candidate subsets of linear models predicting the fecal microbiota Observed index based on all-subset regression.

Rank	SOC	TP	logLik	AICc	Weight
1	−0.438	0.398	−70.807	150.368	0.989
2	−0.399	NA	−76.775	159.994	0.008
3	NA	0.354	−77.915	162.275	0.003

Note: SOC: Soil Organic Carbon; TP: Total Phosphorus; NA indicates that the predictor variable was not selected in that specific sub-model by the dredge function. The values under the predictor columns (SOC, TP) are the standardized regression coefficients for models where they were included; a negative sign indicates an inverse relationship with the Observed index. logLik: log-likelihood of the model; AICc: corrected Akaike Information Criterion, where lower values indicate a better balance between model fit and complexity; weight: Akaike weight, representing the relative probability that the model is the best among the candidate set.

**Table 4:** Linear model constructed from the best candidate for predicting the fecal microbiota Observed index.

Module No.	Model’s Explanatory Power (Deviance R^2^)	Model *p*-Values	Parameter	Std. Coefficient	Std. Coefficient 95% CI	*t*	*p*-Values	Significant
Model-1 (SOC + TP)	0.3154	2.985 × 10^−5^	SOC	−0.44	[−0.66, −0.21]	−3.91	0.000257	***
			TP	0.4	[0.17, 0.62]	3.55	0.00081	***

Note: SOC: Soil Organic Carbon; TP: Total Phosphorus. The model’s explanatory power is represented by the Deviance R^2^. Model *p*-value indicates the overall significance of the regression. Std. coefficient refers to the standardized regression coefficient, with its 95% confidence interval (CI) provided. The *t*-statistic and its corresponding *p*-value are shown for each predictor. Significance levels: *** (*p*-values < 0.001).

**Table 5:** Candidate subsets of linear models predicting the fecal microbiota Shannon index based on all-subset regression.

Rank	SOC	TP	PD	logLik	AICc	Weight
1	0.255	−0.411	NA	−73.300	155.354	0.377
2	0.364	−0.274	0.227	−72.377	155.907	0.286
3	0.476	NA	0.427	−73.988	156.732	0.189
4	NA	−0.435	NA	−75.698	157.840	0.109
5	NA	−0.451	−0.027	−75.679	160.113	0.035

Note: SOC: Soil Organic Carbon; TP: Total Phosphorus; PD: Particle Density. NA indicates that the predictor variable was not selected in that specific sub-model by the dredge function. The values under the predictor columns (pH, TP, PD) are the standardized regression coefficients for models where they were included; a negative sign indicates an inverse relationship with the Shannon index. logLik: log-likelihood of the model; AICc: corrected Akaike Information Criterion, where lower values indicate a better balance between model fit and complexity; weight: Akaike weight, representing the relative probability that the model is the best among the candidate set.

**Table 6:** Linear models constructed from the top 3 candidates for predicting the gut microbiota Shannon index.

Module No.	Model’s Explanatory Power (Deviance R^2^)	Model *p*-Values	Parameter	Std. Coefficient	Std. Coefficient 95% CI	*t*	*p*-Values	Significant
Model-1 (pH + PD)	0.2539	3.18 × 10^−4^	pH	−0.41	[−0.65, −0.18]	−3.513	0.000895	***
			PD	0.25	[0.02, 0.49]	2.177	0.033762	*
Model-2 (pH + TP +PD)	0.2773	5.09 × 10^−4^	pH	−0.27	[−0.59, 0.04]	−1.757	0.0846	
			TP	0.23	[−0.12, 0.57]	1.322	0.1918	
			PD	0.36	[0.08, 0.65]	2.552	0.0136	*
Model-3 (TP + PD)	0.236	6.10 × 10^−4^	TP	0.43	[0.17, 0.69]	3.28	0.001805	**
			PD	0.48	[0.21, 0.74]	3.655	0.000577	***

Note: pH: soil pH; PD: Particle Density; TP: Total Phosphorus. The model’s explanatory power is represented by the Deviance R^2^. Model *p*-value indicates the overall significance of the regression. Std. coefficient refers to the standardized regression coefficient, with its 95% confidence interval (CI) provided. The *t*-statistic and its corresponding *p*-value are shown for each predictor. Significance levels: *** (*p*-values < 0.001), ** (*p*-values < 0.01), * (*p*-values < 0.05).

**Table 7:** Candidate subsets of linear models predicting the fecal microbiota Faith’s PD index based on all-subset regression.

Rank	SOC	TP	logLik	AICc	Weight
1	−0.436	0.332	−72.614	153.983	0.943
2	−0.402	NA	−76.671	159.786	0.052
3	NA	0.289	−79.265	164.975	0.004

Note: SOC: Soil Organic Carbon; TP: Total Phosphorus. NA indicates that the predictor variable was not selected in that specific sub-model by the dredge function. The values under the predictor columns (SOC, TP) are the standardized regression coefficients for models where they were included; a negative sign indicates an inverse relationship with the PD index. logLik: log-likelihood of the model; AICc: corrected Akaike Information Criterion, where lower values indicate a better balance between model fit and complexity; weight: Akaike weight, representing the relative probability that the model is the best among the candidate set.

**Table 8:** Linear model constructed from the best candidate for predicting the fecal microbiota Faith’s PD index.

Module No.	Model’s Explanatory Power (Deviance R^2^)	Model *p*-Values	Parameter	Std. Coefficient	Std. Coefficient 95% CI	*t*	*p*-Values	Significant
Model-1 (SOC + TP)	0.2713	1.658 × 10^−4^	SOC	−0.436	[−0.67, −0.16]	−3.765	0.000407	***
			TP	0.332	[0.10, 0.57]	2.873	0.005759	**

Note: SOC: Soil Organic Carbon; TP: Total Phosphorus. The model’s explanatory power is represented by the Deviance R^2^. Model *p*-value indicates the overall significance of the regression. Std. coefficient refers to the standardized regression coefficient, with its 95% confidence interval (CI) provided. The *t*-statistic and its corresponding *p*-value are shown for each predictor. Significance levels: *** (*p*-values < 0.001), ** (*p*-values < 0.01).

**Table 9:** Multiple regression on distance matrices (MRM) models testing the effects of soil physicochemical properties on β-diversity of gut and soil microbial communities.

Module No.	Model R^2^ Value	Model *p*-Values	Predictor	Coef	*p*-Values
Bray–Curtis (fecal samples) = pH + TP + ExMg	0.2403	0.001	**pH**	0.3083	0.001
			**TP**	0.227	0.003
			ExMg	0.0944	0.0511
Bray–Curtis (soil samples) = ExMg + ExNa	0.3507	0.001	**ExMg**	0.5275	0.001
			ExNa	0.0966	0.2603

Note: The table presents the results of two independent MRM analyses. For each microbial community (gut or soil), a model was constructed using the Bray–Curtis distance matrix (representing β-diversity) as the response variable and the Euclidean distance matrices of selected soil properties as predictors. Model R^2^ indicates the proportion of variation in community dissimilarity explained by the model. Coef represents the standardized regression coefficient; a positive value denotes that greater environmental distance is associated with greater community dissimilarity. Predictor significance levels: bolded predictors are significant (*p* < 0.05). Predictor abbreviations: TP, total phosphorus; ExMg, exchangeable magnesium; ExNa, exchangeable sodium.

## Data Availability

The data presented in this study are openly available in [National Genomics Data Center, China] at [https://ngdc.cncb.ac.cn, accessed on 11 January 2026], reference number [PRJCA049756].
